# Buried chloride stereochemistry in the Protein Data Bank

**DOI:** 10.1186/s12900-014-0019-8

**Published:** 2014-09-23

**Authors:** Oliviero Carugo

**Affiliations:** 1 Department of Structural and Computational Biology, Max F. Perutz Laboratories, Vienna University, Vienna, Austria; 2 Department of Chemistry, University of Pavia, Pavia, Italy

## Abstract

**Background:**

Despite the chloride anion is involved in fundamental biological processes, its interactions with proteins are little known. In particular, we lack a systematic survey of its coordination spheres.

**Results:**

The analysis of a non-redundant set (pairwise sequence identity?<?30%) of 1739 high resolution (<2 Å) crystal structures that contain at least one chloride anion shows that the first coordination spheres of the chlorides are essentially constituted by hydrogen bond donors. Amongst the side-chains positively charged, arginine interacts with chlorides much more frequently than lysine. Although the most common coordination number is 4, the coordination stereochemistry is closer to the expected geometry when the coordination number is 5, suggesting that this is the coordination number towards which the chlorides tend when they interact with proteins.

**Conclusions:**

The results of these analyses are useful in interpreting, describing, and validating new protein crystal structures that contain chloride anions.

## Background

It is sufficient to open any biochemistry or bioinorganic chemistry book to verify that, although chloride is essential for any form of life, its biological chemistry receives less attention than other small ions like for example sodium(I), calcium(II) or magnesium(II).

Together with sodium(I) and potassium(I) ions, chloride is responsible for the osmotic and charge stability of cells [1]. Chloride is essential to maintain cellular and whole body pH, which is mainly buffered by the CO_2_/HCO_3_^?^ equilibrium, since the exchange of bicarbonate across the plasma membrane is coupled with chloride exchange [2]. A well-known channel with specificity for anions, in particular chloride and bicarbonate, is the cystic fibrosis transmembrane conductance regulator [3].

Chloride is also present in some proteins. For example in many amylases, a chloride anion coordinated by arginine and lysine side-chains is bound close the active site, where it may assist the reaction [4]. In photosystem II two chloride anions have been identified close to the Mn_4_CaO_6_ cluster and may contribute to it stability/reactivity [5],[6]. The two chloride binding sites are at the start of hydrogen bond networks that connect the cluster with the bulk solution. These networks may function as proton exit channels or water inlet channels [5],[6].

In this manuscript, the chloride anions observed in the protein crystal structures deposited in the Protein Data Bank [7],[8] are analyzed systematically, with the aim of finding their preferred coordination numbers, the amino acids/atoms that tend to interact with them, and their coordination stereochemistry. The results of these analyses should prove useful as benchmarks against which it is possible to critically validate new experimental results.

## Results and discussion

### Solvent exposure of the chlorides

The solvent accessibility of a chloride anion is defined here as the fraction of surface that is accessible to a solvent probe (radius?=?1.4 Å) and that is not already covered by other atoms, including water molecules that were experimentally positioned. This is an uncommon definition of solvent accessibility. However, it is adopted here to identify the chloride anions with a well defined first coordination sphere.

**Figure 1:**
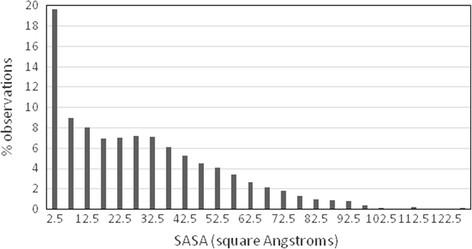
Distribution of the SASA values of the chloride anions observed in a non-redundant set of chloride-containing protein crystal structures.

On average, the chloride anions have 29 Å^2^ exposed to the solvent, which means about 22% of their surface (the surface of a sphere of radius equal to 1.81?+?1.40 Å is 129 Å^2^). Several chloride anions are well packed (Figure 1). Nearly 20% of them have a residual SASA lower than 5 Å^2^. The attention was focused on them. The other chloride anions, located at the protein surface and considerably exposed to the solvent were disregarded since their first coordination sphere is incomplete.

Quite frequently two or more very similar chloride anions are present in an individual PDB file, for example, when one of them interacts with a polypeptide chain and another interacts with another polypeptide chain of identical sequence. These chloride anions are in the same asymmetric unit and are therefore not equivalent from a symmetry perspective. However, they are chemically analogous and therefore only one of them must be considered to avoid data redundancy. For this reason, only one chloride anion (the first in the list) was analyzed in each PDB file, though this implies that some chloride anions are arbitrarily disregarded.

### Coordination numbers

The distance between a chloride anion and the atoms that are in its first coordination sphere is within 3.4 Å. Chloride coordination by urea derivatives, molecules that mimic polypeptides, has been studied in detail and it has been observed that the distances between the chloride anions and the urea nitrogen atoms range from 3.26 to 3.35 Å [9]. In chloride supramolecular salts of diammonium-bis-pyridinium cations, the distances between the chloride anions and the cationic nitrogen atoms range from 3.03 to 3.04 Å [10].

**Figure 2:**
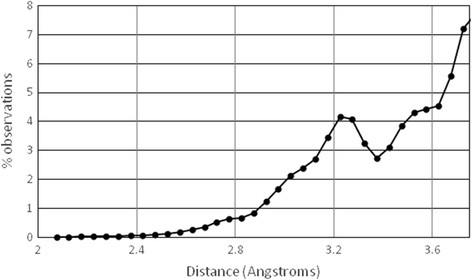
Distribution of the distances between the buried chloride anion and the atoms around it observed in a non-redundant set of chloride-containing protein crystal structures.

In the data set of the chloride-containing PDB files that are examined here, it is possible to examine these distances from a statistical perspective. Figure 2 shows the distribution of the distances between the chloride anion and the atoms (any type of atom) around it. Obviously, there are nearly no atoms within 2.5 Å, and the number of atoms increases at larger distances. However, this increase is discontinuous. There is a maximum around 3.2 Å, followed by a minimum around 3.4 Å.

This type of trend indicates a considerable clustering tendency of the atoms around the chloride anions, according to the Lacey-Cole statistics, which is routinely used (i) to verify if the objects are randomly distributed or have a natural tendency to cluster into well separate groups and (ii) to estimate the natural separation between adjacent groups [11]. In other words, the results shown in Figure 2 suggest that (i) the atoms are not randomly distributed around chloride anions (at any distance from the anion) and that (ii) the atoms that surround a chloride anion tend to be within 3.4 Å from the chloride anion.

The atoms are not randomly distributed but tend to be within a sphere of radius 3.4 Å, centred on the chloride anion.

**Figure 3:**
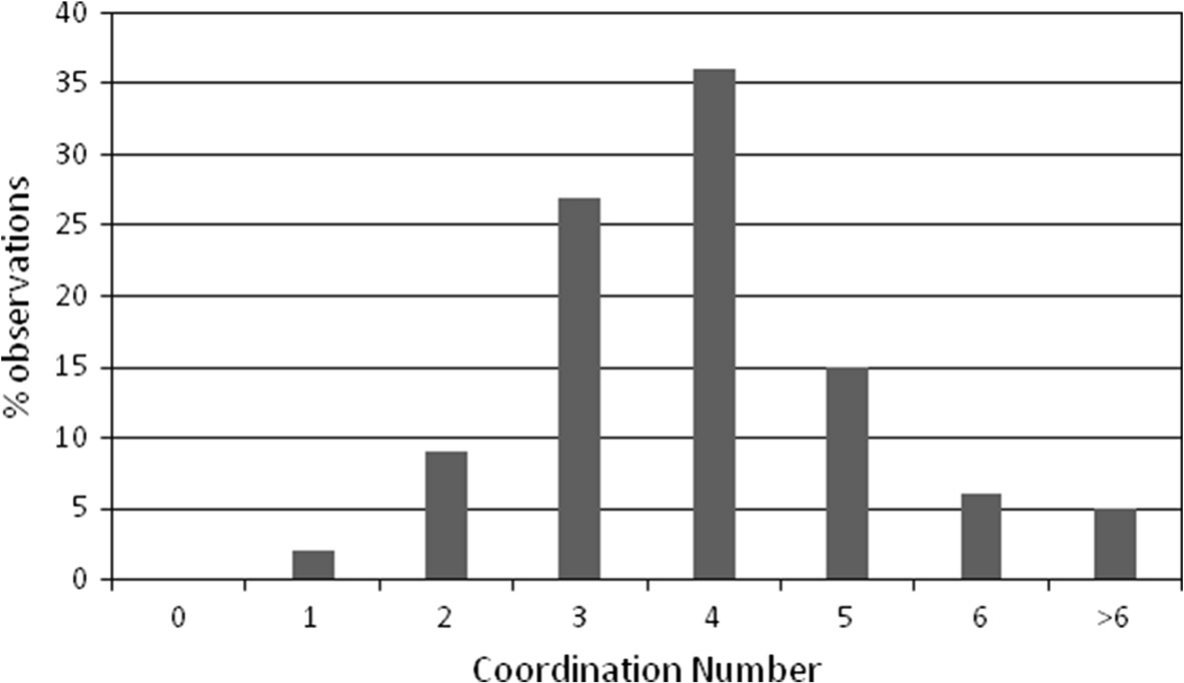
Distribution of the coordination numbers of the buried chloride anions observed in a non-redundant set of chloride-containing protein crystal structures.

On the basis of these observations, an atom was considered to be within the first coordination sphere of a chloride anion if it falls in a sphere of radius equal to 3.4 Å. Figure 3 shows the distribution of the coordination numbers. The most common coordination numbers are 3, 4, and 5. Only about 10% of the chlorides have a coordination number lower than 3 or higher than 5.

**Figure 4:**
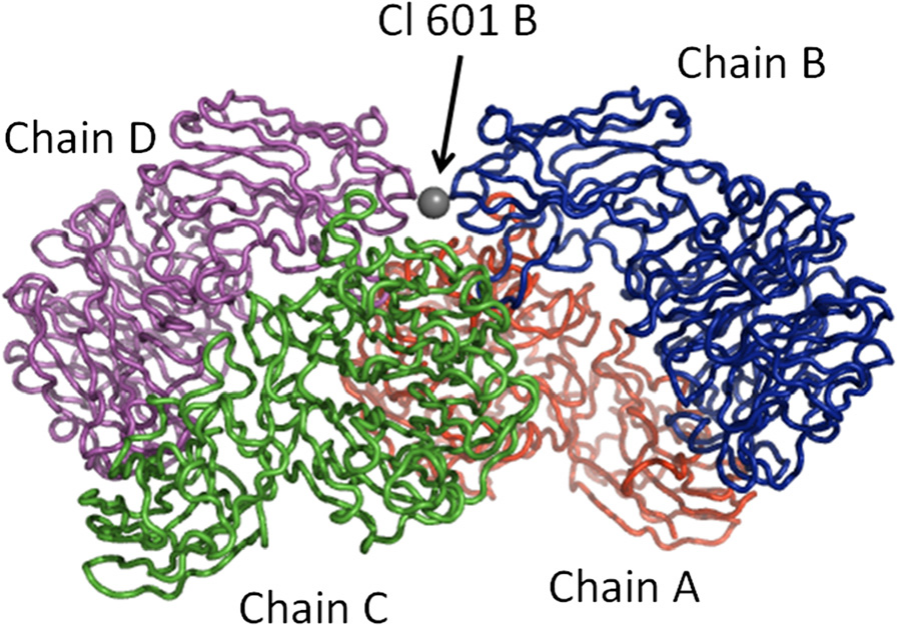
**Structure of a glycoside hydrolase from** ***Parabacteroides distasonis ATCC 8503*** **with a «naked» chloride anion (PDB identification code 4fj6).**

Only one case of coordination number equal to 0 was observed (Figure 4): it is the atom Cl 601 B, located in between the chains B and D of a tetrameric glycoside hydrolase from *Parabacteroides distasonis ATCC 8503* (PDB file 1fj6), which is naked, in the sense that its closest neighbour is the main chain nitrogen atom of Lys 50 B, at 3.48 Å [12].

The atomic displacement parameters (ADPs), normalized to zero mean and unit variance (as it is usually done when it is necessary to compare B-factors of different protein crystal structures [13],[14]), of the chloride anions and their donor atoms are independent of the coordination number and do not present any particular trend. The average ADP of the chloride anions is slightly larger than zero (0.10), while the average ADP of the ligands is slightly smaller than zero for protein donor atoms (?0.40) and for donor atoms of small molecules (?0.16); it is on the contrary slightly larger than zero for water molecules coordinated to the chlorides (0.10).

### Residues in the first coordination sphere

Table 1 summarizes the frequencies of each type of residue (including water) in the first coordination sphere of the chloride anions.

**Table 1:** Percentage with which the various types of amino acids are found in the first coordination sphere of chloride anions, for various coordination numbers (CN)

**Residue**	**CN?=?2**	**CN?=?3**	**CN?=?4**	**CN?=?5**	**CN?=?6**
Ala	3.5	1.5	2.7	2.7	4.2
Arg	17.4	11.9	13.2	10.9	12.5
Asn	2.3	6.5	6.6	5.6	6.2
Asp	4.7	3.4	1.8	5.0	0.0
Cys	0.0	1.0	0.3	1.5	0.0
Gln	4.7	4.8	2.7	3.0	2.8
Glu	4.7	2.7	3.0	2.4	6.2
Gly	4.7	2.7	4.8	6.2	6.2
His	9.3	5.1	3.1	5.0	4.2
Ile	0.0	1.7	1.8	1.5	1.4
Leu	1.2	2.7	1.9	2.4	4.9
Lys	3.5	5.8	3.1	3.0	2.8
Met	0.0	0.5	0.6	0.3	2.1
Phe	0.0	1.7	0.9	0.9	2.8
Pro	0.0	0.2	0.3	1.5	0.7
Ser	9.3	8.2	7.2	9.8	3.5
Thr	4.7	6.3	5.7	3.3	4.9
Trp	2.3	1.2	1.3	0.9	2.8
Tyr	1.2	4.4	3.3	1.5	1.4
Val	3.5	3.6	1.5	1.5	3.5
HOH	23.0	24.1	34.2	31.1	26.9

Water is very frequent, between one fourth and one third, indicating that it is rare that chlorine interacts only with atoms of protein.

Arginine is also very frequent, in the range 11-17%, considerably more than expected ¿ only about 6% of the amino acids are arginines in proteins [15]. Also histidine is more frequent than expected: 3-9% of the residues in the chloride first coordination sphere are histidines, while only about 2-3% of the residues are histidines in proteins [15]. Curiously, lysine is less frequent, in the range 3-6%, roughly as it is expected (about 6% of the residues are lysines in proteins [15]).

Polar residues (Asn, Gln, Ser, and Thr) are also quite frequent, in the range 17-26%. Glycine is also quite frequent, in the range 3-6%, as expected [15].

Despite the electrostatic repulsion between their anionic side-chains and the chloride anion, aspartate and glutamate are not totally absent (range 6-9%). Usually they contact the chloride anion with their main-chain nitrogen atom and this interaction is reinforced if the chloride anion interacts also with cations, which can be coordinated by the carboxylate moiety of Glu and Asp. The chloride coordination via the main-chain amido group explains also the presence of apolar residues in the chloride first coordination sphere (range 11-24%).

### Atoms in the first coordination sphere

The large majority of the atoms in the chloride first coordination sphere are oxygen and nitrogen atoms (Table 2). At lower coordination numbers, nitrogen atoms are considerable more frequent than oxygen atoms, while the two types of elements have comparable frequencies at higher coordination numbers. Other types of chemical elements, typically carbon, are present in considerable amount only when the coordination number is high. For example, only 4% of the atoms in the first coordination sphere are carbons when the coordination number is 2, while up to 20% of the atoms in the first coordination sphere are carbons when the coordination number is 6.

**Table 2:** Percentage with which the various types of chemical elements are found in the first coordination sphere of chloride anions, for various coordination numbers (CN)

**Element**	**CN?=?2**	**CN?=?3**	**CN?=?4**	**CN?=?5**	**CN?=?6**
N	61.1	56.0	46.6	38.9	39.1
O	34.4	40.4	48.3	47.3	37.9
C	4.4	3.3	4.7	12.7	20.1
Others	0.0	0.2	0.4	1.1	2.9

In general, the presence of carbon atoms in the first coordination sphere of a chloride anion may seem surprising, since most of the carbon atoms that can be present in protein crystals are rather apolar and therefore little prone to interact with a charged ion. However, the fact that carbon atoms are rather abundant only at high coordination numbers suggests that in many cases their presence is purely accidental. They are in general covalently bound to another atom that can be attracted electrostatically by the chloride anion, for example, a carboxylic carbon atom of the side-chain of an asparagine, which is bound to an amide NH2 moiety, which in turn may form a hydrogen bond with the chloride anion.

A deeper analysis of the composition of the first coordination sphere of the chloride anions was conducted only on the protein atoms, excluding cofactors/prosthetic-groups, since only for the protein atoms it is easy to classify automatically the type of electronic structure and covalent context of each non-hydrogen atom.

Table 3 shows a brief summary of the results. As expected, most of the atoms of the chloride first coordination sphere are potential hydrogen bond donors: the main-chain nitrogen atom, other N-H groups of the side chains of some residues (glutamine, asparagine, histidine, and tryptophan), ammonium and guanidinium groups of lysine and arginine, and O-H groups of the side-chains of some amino acids (serine, threonine, and tyrosine).

**Table 3:** Percentage with which the various types of protein atoms are found in the first coordination sphere of chloride anions, for various coordination numbers (CN)

**Atom**	**CN?=?2**	**CN?=?3**	**CN?=?4**	**CN?=?5**	**CN?=?6**
Main-chain N-H	47.0	39.0	35.2	33.6	27.6
Other N-H	13.6	16.7	14.6	8.6	12.5
N charged	19.7	18.9	22.3	15.5	17.2
Main-chain O	1.5	3.5	3.6	8.6	9.5
O-H	10.6	15.0	15.5	12.1	5.8
C?=?O	3.0	2.6	0.9	2.6	3.8
Charged O	0.0	0.0	0.7	1.3	1.9
Main-chain C	0.0	0.3	0.7	2.6	1.0
CA	0.0	0.0	1.8	4.7	3.8
CB	0.0	1.3	1.1	4.3	6.7
Other C	4.5	2.4	3.5	5.5	9.6
S	0.0	0.3	0.2	0.0	1.0

Unexpected atoms are also observed in the chloride first coordination spheres (carbon atoms and even negatively charged oxygen atoms of the side chains of aspartate and glutamate). However, their presence is particularly evident only at high coordination numbers and it is reasonable to suppose that their presence in the chloride first coordination sphere is simply accidental.

### Analysis of the coordination polyhedra

The interactions between the chloride anion and the atoms that surround it are expected to be essentially electrostatic and thus non-directional. Consequently, it is possible to predict the most stable conformation for each coordination number. When two, identical atoms are in contact with the chloride anion, they should occupy two positions diametrically opposite on a sphere centred on the chloride anion. This results in a linear stereochemistry with an angle X-Cl-X equal to 180 degrees.

Analogously, if the coordination number is three, the stereochemistry is expected to be trigonal planar with the chloride lying at the centre of an equilateral triangle, at the vertices of which there are three, identical atoms. There are then three, identical angles X-Cl-X equal to 120 degrees.

If the coordination number is equal to four, two, nearly degenerate stereochemistries are possible. In the tetrahedral stereochemistry, the chloride anion is at the centre of a tetrahedron, the vertices of which are occupied by four, identical atoms; there are then six X-Cl-X angles, all equal to 109.5 degrees. In the square planar stereochemistry, four, identical atoms are at the vertices of a square, at the centre of which lies the chloride anion; in this case there are then two X-Cl-X angles of 180 degrees and four X-Cl-X angles of 90 degrees.

Also for the coordination number five, two stereochemistries have comparable energies. In the trigonal bipyramidal stereochemistry, two trigonal pyramids share a triangular face and the five vertices are occupied by five, identical atoms, while the chloride anion lies in the centre of the polyhedron. There are then ten X-Cl-X angles, one of which is equal to 180 degrees, three of which are equal to 120 degrees, and six of which are equal to 90 degrees. In the alternative, square pyramidal stereochemistry, the chloride anion lies at the centre of the square base of the pyramid and five, identical atoms occupy the vertices of the polyhedron. Two of the ten X-Cl-X angles are then equal to 180 degrees while all the other eight are equal to 90 degrees.

If the coordination number is equal to six, the most stable stereochemistry is octahedral, with six, identical atoms at the vertices of the octahedron and the chloride anion at the centre of the octahedron. Three of the 15 X-Cl-X angles are then equal to 180 degrees, while all the other 12 are equal to 90 degrees. Another possible stereochemistry is the trigonal prismatic (or anti-prismatic), where the chloride anion is at the centre of a trigonal prism, the vertices of which are occupied by six, identical atoms. In this case, the amplitudes of the X-Cl-X angles have not fixed values; the only constraint is that six of them must be equal to each other and that the other nine must be equal to each other, with the two amplitudes being independent of each other.

Obviously, deviations from these ideal stereochemistries are possible. They might be due to the fact the atoms that surround the chloride anion are not identical and can have different dimensions. It is also necessary to consider that the interactions between the chloride anion with some of its neighbours can be different from the interactions with other neighbours. In addition, one cannot ignore the sterical role of the chemical groups that may influence the atoms of the first coordination sphere even if they do not interact directly with the chloride anion.

The degree of the distortion from the ideal geometries was measured by means of the distortions of the angles centred on the chloride anion. For example, for a chloride anion surrounded by two atoms X and Y, with the angle X-Cl-Y being equal to A, the quantity D?=?|A-180| was computed and the stereochemistry was considered to be linear if D?<?20 degrees. For a chloride anion surrounded by the three atoms X, Y, and Z, the amplitudes of the three angles X-Cl-Y (indicated by A1), X-Cl-Z (A2), and Y-Cl-Z (A3) was computed and the stereochemistry was considered to be trigonal planar if the quantity D?=?(|A1-120|?+?|A2-120|?+?|A3-120|)/3 was less than 20 degrees.

An analogous quantity D was computed for larger coordination numbers, by considering that different angle permutations are possible and must then be inspected. For example, in a square planar stereochemistry, two angles centred on the chloride anion must be equal to 180 degrees and four angles must be equal to 90 degrees. There are numerous ways to write this list of these six angle amplitudes: one might be 180, 180, 90, 90, 90, 90; a second might be 180, 90, 180, 90, 90, 90; a third might be 180, 90, 90, 180, 90, 90; and so on. In total, there are 15 permutations, and all of them must be inspected to measure the minimal distortion from the ideal, square planar stereochemistry of a real molecular moiety constituted by a chloride anion surround by four atoms. Analogously, there are 45 permutations of the angles centred on the chloride anion in a square planar geometry, 840 in a trigonal bipyramidal geometry, 455 in a octahedral geometry, and 5005 in a trigonal prismatic geometry.

The analysis of the deviations of the real stereochemistry around the chloride anions from the ideal stereochemistries is rather surprising (Table 4). The cases with coordination numbers two are severely distorted, on average, from the ideal stereochemistry: the average distortion is larger than 70 degrees. This clearly suggests that the second coordination sphere play a relevant role and, in other words, that the coordination number two is often just a misinterpretation of the structural data: other atoms should have been included into the first coordination sphere, despite they are too distant from the chloride anion or despite their positions were not determined with sufficient accuracy.

**Table 4:** Distortions from the ideal geometries of the chloride first coordination spheres

**Coordination number**	**Stereochemistry**	**Average distortions with standard error in parentheses (degrees)**	**% of chlorides that assume the ideal stereochemistry (within 20 degrees)**
2	Linear	75.0 (5.4)	0
3	Trigonal	27.5 (1.1)	29
4	Tetrahedral	25.8 (0.7)	25
	Square planar	27.5 (0.6)	10
5	Trigonal bipyramidal	18.4 (0.6)	70
	Square pyramidal	24.7 (0.7)	21
6	Octahedral	34.8 (0.9)	0
	Trigonal prismatic	30.6 (1.1)	7

The distortions from the ideal geometry are less pronounced for coordination numbers higher than two. They are still relatively large for coordination number three (27 degrees), four (26¿27 degrees) and six (31 degrees) and they are smaller for coordination number five (18¿25 degrees). Notably, nearly all (91%) the chlorides surrounded by five atoms can be described, within 20 degrees, as regular trigonal bipyramids or square pyramids. A so high percentage is rather unexpected, since the actual coordination sphere of a chloride anion inaccessible to the solvent is expected to be influenced severely by the overall molecular packing requirements. In other words, the type and the shape of the molecules and of the molecular fragments that can be present in a protein crystal are expected to restrain the natural tendency to occupy optimally the space, because of their covalent constraints. It is then possible to hypothesize that five is the more appropriate coordination number for a chloride anion in a protein crystal structure. In other words, a chloride anion seems to have a natural tendency to surround itself with five atoms, when it co-crystallizes with proteins and other molecules that might be present in the crystallization medium.

### Packing bridges involving chlorides

About 10¿20% of the chlorides examined in the present paper are bound to residues of different polypeptide chains, behaving as packing bridges [16]. This percentage varies with the coordination number: it is 9% for coordination number 2, 21% for 3, 19% for 4, 12% for 5, and 7% for 6. About one-half of these packing bridges connect polypeptide chains in the same asymmetric unit and the other half connect chains that are related by a symmetry operation in the crystallized material.

This shows that the chloride anions share a feature with other ions and small molecules: they have a marked tendency to connect adjacent molecules in the solid state, stabilizing nascent crystals. Given that several charged and polar residues form the surface of globular proteins, it is not surprising that chloride anions can approach the protein surface in solution and then connect a protein molecule with an adjacent protein in the nucleation phase of the crystallization.

## Conclusions

A non-redundant set (pairwise sequence identity?<?30%) of 1739 high resolution (<2 Å) crystal structures that contain chloride anions was analyzed. The first coordination sphere of the chlorides is essentially constituted by hydrogen bond donors: main-chain N-H groups and side-chains N-H and O-H groups. Amongst the side-chains positively charged, arginine interacts with chlorides much more frequently than lysine. The most common coordination number is 4, though the coordination stereochemistry is closer to the expected geometry when the coordination number is 5, suggesting that this is the coordination number towards which the chlorides are predisposed when they interact with proteins.

These results should prove useful to examine new protein crystal structures that contain chloride anions, especially to validate, on the basis of the chemical and geometrical features, the chloride binding sites.

## Methods

All the files of the Protein Data Bank that contain at least one chloride ion were identified with help of the server PLI (http://bioinformatics.istge.it/pli/; [17]) and only the crystal structures refined at a resolution better than 2.0 Å were retained. Sequence redundancy was reduced to 30% of pairwise sequence identity with the service provided by the PDB advanced search service and 1739 PDB files were retained (see Additional file 1).

It is important to observe that this dataset probably underestimate the number of chlorides anions in protein crystal structures. It was in fact observed that chlorides and other ions, for example phosphate and metal cations, tend to be overlooked in macromolecular crystallography and misinterpreted as water molecules, unless soft x-rays diffraction data are used [18]. However, routinely, these data are not collected yet. However, if it is true that there are some false negatives (chlorides misinterpreted as water molecules) it is also true that at high resolution there should be few false positives (electron density peaks that are interpreted as chlorides despite they are not chlorides). Moreover, the data refined with soft X-rays are still not sufficiently numerous to allow a statistical survey of the chloride coordination chemistry in biological macromolecules.

The solvent accessible surface areas were computed with Naccess (http://www.bioinf.manchester.ac.uk/naccess/), with a probe radius of 1.4 Å, in the modality that includes the hetero-atoms in the computations. The Shannon¿s ionic radius of the Chloride (1.81 Å) was used [19]. The solvent accessibility of a chloride anion is the fraction of surface that is accessible to a solvent probe and that is not already covered by other atoms, including water molecules that were experimentally positioned.

Packing contacts and packing bridges were identified as described previously [16].

## Competing interests

The author declares that he has no competing interests.

## Author¿s contributions

OC designed and executed the project and wrote the manuscript.

## Additional file

## Supplementary Material

Additional file 1: 1739 high resolution (< 2 Angstroms) crystal structures that contain chloride anions. Click here for file
