# Supplementary material for: Buried chloride stereochemistry in the Protein Data Bank
Source: BMC Struct Biol. 2014 Sep 23;14:19. doi: 10.1186/s12900-014-0019-8 (PMC4582432; doi:10.1186/s12900-014-0019-8)
Supplement: Additional file 1: — 1739 high resolution (< 2 Angstroms) crystal structures that contain chloride anions. [file s12900-014-0019-8-S1.docx]

Supplementary material: 1739 high resolution (< 2 Angstroms) crystal

structures that contain chloride anions

1a7d 1a7w 1a9x 1aa5 1ag9 1av2 1b0u 1bes 1brt 1bxe 1byz 1c0q

1c0r 1c1k 1c3q 1c4d 1cb6 1chp 1ct9 1cxp 1czq 1d2a 1da3 1dg6

1dj0 1dl5 1dp4 1dqs 1dqt 1dw9 1dyp 1e3a 1e42 1e58 1e8c 1eaq

1ekj 1el4 1enn 1eon 1esl 1eu8 1ezw 1f0l 1f1e 1f7l 1fm0 1g29

1g4i 1g7a 1g9j 1ga1 1gut 1gxt 1gyh 1h80 1hbn 1hhf 1hx6 1hxn

1hyu 1i7w 1icg 1id9 1idw 1iee 1iha 1ikp 1is9 1j0h 1j9m 1jak

1jb7 1jdp 1jfx 1jif 1jt1 1jzt 1k04 1k12 1k2x 1k5c 1kea 1kjq

1knq 1kq3 1kwg 1kww 1kx5 1l4x 1lfa 1lri 1ltz 1lvw 1lyv 1m55

1m6s 1mj5 1mof 1mwq 1n0w 1n2z 1n7z 1na0 1nc7 1nnl 1nqj 1nw6

1nyc 1nzd 1o1h 1o54 1o6t 1ods 1ogd 1oj7 1ok0 1onw 1oru 1oyj

1pfb 1pfe 1pg4 1pn0 1pu6 1pyz 1q4t 1q7r 1q8c 1qb0 1qbz 1qd8

1qdc 1qh3 1qh8 1qhd 1qhq 1qq7 1qul 1qz9 1r55 1r66 1r6j 1r89

1rdo 1rhc 1rki 1rkq 1rm6 1rxi 1ry9 1rya 1s55 1s7f 1sen 1sf9

1sfx 1sgw 1sho 1skz 1so7 1svf 1sw5 1sx7 1szd 1t0h 1t3c 1t6c

1tef 1tfj 1tgg 1tif 1tjb 1tmx 1tzy 1u55 1ue2 1ufy 1uhx 1uhy

1urm 1urn 1uut 1uwv 1uy0 1v3w 1v3z 1v6p 1v7w 1v8c 1va0 1vch

1vcl 1vct 1vjf 1vjl 1vkh 1vlb 1vly 1vme 1vpd 1vph 1vqq 1vr5

1vzi 1w07 1w15 1w23 1w3m 1w4s 1w5m 1w5u 1w7c 1w9h 1wcv 1whz

1wky 1wl8 1wlu 1wpb 1wrv 1wv3 1wvf 1wvv 1x6v 1xg0 1xgk 1xki

1xmk 1xov 1xpe 1xv5 1y4m 1y88 1y8z 1yd6 1yf9 1yfu 1yq2 1yve

1z2u 1z40 1z47 1z70 1zch 1zd6 1zei 1zej 1ziz 1zkp 1zmk 1zn8

1zuk 1zv7 2a0m 2a2c 2a2k 2a53 2a65 2a8j 2a9s 2abs 2acy 2aee

2ag2 2agk 2ah2 2al1 2anv 2aqj 2arz 2av4 2awp 2axw 2b0v 2b3h

2b8m 2bb6 2bfd 2bi0 2bjd 2bjk 2bk9 2bry 2bsj 2bu3 2bwr 2c0g

2c2n 2c3m 2c43 2c5g 2cc6 2cdo 2cf7 2cpg 2cz4 2cz9 2d1s 2d2e

2d8d 2dc4 2dct 2dho 2djf 2dpq 2dsj 2dur 2duy 2dv3 2dxa 2dya

2e1f 2e3w 2e4t 2e6u 2ebo 2ecs 2eh9 2eht 2ess 2etj 2etv 2evr

2f46 2f4m 2f7b 2fa5 2fct 2fcx 2fd0 2fij 2fj9 2fk5 2fms 2fom

2fpu 2ftr 2fu4 2fvv 2fy6 2fyq 2fzv 2g4k 2g4l 2g4r 2g8f 2g8i

2g9f 2g9w 2g9z 2gf3 2gff 2gfh 2gfn 2ghs 2giv 2gkr 2gnq 2go3

2gs3 2gvk 2gwn 2h00 2h98 2h9f 2haw 2hba 2hc1 2hcf 2hek 2heu

2hhz 2hi0 2hiy 2hjp 2hkv 2hl5 2hls 2hox 2hq7 2hq9 2hqk 2hs1

2htd 2hxv 2i02 2i0q 2i0z 2i3d 2i6g 2i6h 2i7q 2i8d 2i9f 2i9w

2iay 2icg 2id3 2id8 2ifx 2ihd 2iqy 2is9 2ism 2ist 2it9 2iw0

2iwk 2iyv 2izr 2j07 2j1n 2j46 2j7u 2j91 2j9a 2ja2 2jaf 2jd4

2je6 2je8 2jfr 2jh1 2jlq 2jlv 2nl9 2nlv 2nns 2no4 2nq3 2nrt

2nt4 2nte 2ntt 2nwh 2nxw 2o0r 2o1q 2o20 2o4v 2o62 2o6f 2o6p

2o7a 2o8n 2o9s 2oa9 2oaf 2obp 2od4 2od5 2odi 2og6 2ogi 2oik

2okf 2okg 2olr 2olt 2omd 2oq0 2oqb 2oqm 2oso 2ou3 2oui 2ox0

2ozn 2p02 2p09 2p0a 2p0e 2p0k 2p0n 2p0w 2p2n 2p2v 2p2y 2p5v

2p67 2p7i 2p8i 2p97 2p9w 2pa7 2pbl 2pfg 2pfh 2pfi 2pfx 2pgo

2pke 2plr 2pmu 2pn2 2pos 2pq7 2pqt 2pu3 2puz 2px2 2pyw 2pyx

2q10 2q24 2q2f 2q35 2q3e 2q3x 2q6q 2q7b 2q7x 2q8n 2q9o 2qa9

2qee 2qf7 2qhf 2qih 2qiw 2qjv 2qjw 2qlt 2qml 2qmo 2qnl 2qrj

2qrr 2qsa 2qsk 2qsq 2qt6 2qts 2quy 2qw5 2qx8 2qyc 2r0c 2r11

2r13 2r1i 2r37 2r3b 2r5o 2r6o 2r85 2r8e 2r9f 2rag 2ras 2rbc

2rc8 2rdc 2ree 2rfm 2rfv 2rg1 2rg8 2rhm 2rij 2ril 2rj2 2rje

2rld 2uuy 2ux7 2uya 2v1p 2v1q 2v1x 2v2h 2v3z 2v4a 2v79 2v7f

2v7u 2v8t 2v9k 2v9v 2vay 2vcg 2vd8 2vec 2vfr 2vfx 2vg3 2vj0

2vji 2vjk 2vlg 2vli 2vm9 2vof 2vq2 2vrs 2vun 2vv6 2vve 2vxz

2vyo 2w0r 2w20 2w3q 2w5e 2w70 2w79 2w7z 2w98 2waw 2wb6 2wbf

2wbm 2wcz 2wfj 2wi8 2wj9 2wjq 2wjw 2wka 2wkq 2wkx 2wm3 2wml

2wns 2wny 2wo4 2wpq 2wq0 2wq9 2wta 2wtp 2ww5 2wwp 2wyt 2wzb

2wzn 2x1d 2x3c 2x5g 2x5o 2x5x 2x7b 2x7r 2x8s 2x98 2xb0 2xbp

2xci 2xdh 2xe4 2xep 2xev 2xex 2xfg 2xiw 2xjp 2xmj 2xpw 2xqh

2xsa 2xsq 2xsw 2xu3 2xus 2xvs 2xvx 2xyq 2xzk 2y08 2y2m 2y3g

2y4r 2y5p 2y5s 2y6x 2y75 2y78 2y8u 2yan 2yav 2yd6 2yeo 2yfd

2yfr 2yg9 2yhg 2yhw 2yil 2yo3 2yoi 2ype 2yv5 2yve 2z2n 2z39

2z3j 2z9x 2za4 2zaw 2zdp 2zf9 2zsg 2zy4 2zy6 363d 3a02 3a2a

3a6h 3a6r 3a7k 3ab9 3aei 3ago 3ah7 3ajv 3ajx 3akh 3akq 3aqj

3arc 3arl 3atv 3avr 3b01 3b0g 3b0x 3b1v 3b36 3b72 3b7c 3b8b

3b8l 3b9t 3bc8 3bd1 3bdv 3be6 3bgu 3bhd 3bhq 3bhy 3bit 3bjn

3bkx 3bmb 3bmw 3bny 3bos 3bpk 3bt5 3bwv 3bwx 3bwz 3bxp 3byq

3c18 3c1q 3c3k 3c3p 3c44 3c4z 3c5e 3c61 3c6v 3c7m 3c7t 3c7x

3c8l 3cd3 3chv 3cin 3cj8 3cjd 3ck1 3ck6 3clm 3cmb 3cmg 3cng

3cnv 3cp0 3cqb 3crr 3csh 3cz1 3cz4 3d00 3d02 3d0k 3d0s 3d1p

3d1r 3d32 3d6k 3d7j 3daq 3dci 3dcx 3ddh 3dew 3di4 3dlo 3dnu

3dr8 3dsm 3dtt 3dvv 3dxl 3dyi 3e05 3e15 3e18 3e78 3e8m 3e8y

3e9l 3eby 3eeu 3egv 3el6 3elk 3elq 3els 3enj 3eo7 3er7 3erp

3es1 3es4 3etj 3eto 3eul 3ex6 3exn 3ezw 3f2i 3f3s 3f43 3f4a

3f4m 3f5o 3f5r 3f7q 3f7w 3f95 3faj 3fak 3fbl 3fdb 3fed 3fg1

3fgh 3fh1 3fj1 3fjv 3fla 3fm5 3fo5 3fp3 3fr1 3frq 3ft1 3fvb

3fvy 3fwk 3fxq 3fyb 3fzn 3fzy 3g0t 3g16 3g1z 3g3k 3g5o 3g7q

3g7r 3g7u 3g8z 3ga7 3gae 3gai 3gan 3gc2 3ge3 3geu 3gf3 3gfa

3gg9 3gmf 3gnu 3go5 3goa 3goc 3gr3 3gri 3gsj 3gt5 3gxh 3h05

3h1n 3h4x 3h5f 3h7c 3h8h 3hal 3hb2 3hbn 3hc1 3hdj 3hfw 3hht

3hhy 3hil 3hjb 3hlx 3hmf 3hpc 3hq1 3hra 3hrl 3hup 3hwp 3hww

3hx9 3i0y 3i0z 3i2k 3i36 3i5w 3i6y 3i7u 3i84 3i96 3ia7 3id7

3idv 3ie5 3igs 3ihu 3ihv 3ii2 3iii 3iis 3iix 3ij3 3ij5 3ijw

3imh 3imm 3inz 3ip0 3iq1 3iqu 3ir4 3isa 3isq 3ist 3ite 3iuo

3iux 3iv0 3ivb 3ive 3iwf 3jpo 3jpq 3jps 3jq0 3jq1 3jqy 3ju4

3jx9 3jy6 3jze 3k1t 3k28 3k2a 3k2o 3k4i 3k50 3k5k 3k6m 3k6q

3k7c 3k9d 3ka8 3kan 3kbk 3kbr 3kcp 3kdw 3keb 3keo 3kff 3kg0

3kgw 3kgy 3khi 3ki0 3kkg 3kma 3koq 3kq0 3kqi 3kre 3ks6 3ktc

3kut 3kvh 3kwe 3kwk 3kwu 3kyz 3kzp 3l03 3l12 3l25 3l29 3l2h

3l50 3l5i 3l6t 3lat 3lbe 3lcc 3ldv 3lhe 3lhr 3lid 3lk7 3lkt

3ll1 3llc 3llx 3lqx 3lr4 3luu 3lx4 3lxq 3m07 3m1u 3m1x 3m3h

3m4r 3m4z 3m6j 3m6w 3m6z 3m86 3mbd 3mbh 3mby 3mc1 3mc3 3mc4

3mdo 3mdq 3mhx 3mko 3moz 3mqd 3mqz 3mst 3msu 3msw 3mt0 3muj

3mux 3mvu 3mvx 3mxo 3myx 3mz0 3mz1 3n08 3n0q 3n2b 3n3r 3n5l

3n5w 3n79 3n7h 3n9b 3n9r 3na6 3nce 3ndh 3nh4 3nkl 3nm8 3no2

3no4 3npf 3nq9 3nqi 3nrr 3nt1 3nt6 3nuq 3nvs 3nwn 3nyh 3nyq

3o0h 3o0q 3o1n 3o3y 3o4d 3o5a 3o5v 3o8m 3o9z 3obc 3oc2 3odg

3ofg 3ogh 3ohg 3oin 3oio 3oj6 3omc 3on6 3onp 3oow 3oox 3op9

3oq2 3oqg 3oqp 3or3 3oru 3os4 3ose 3ot2 3oti 3otn 3oug 3ov8

3owv 3oxh 3oyv 3oyz 3p1v 3p3o 3p72 3p8a 3p8k 3pa6 3pdd 3pdf

3pf6 3pf7 3pfe 3pij 3pj0 3pjy 3pl0 3plf 3pm8 3pmc 3pmo 3pnx

3ppm 3pr6 3pua 3pvj 3pyc 3q12 3q18 3q1n 3q2i 3q3u 3q46 3q4s

3q9i 3qk8 3qlj 3qm3 3qnm 3qoo 3qpg 3qr7 3qsg 3qte 3qu5 3qvp

3qvq 3qxb 3qy3 3qzu 3r0n 3r0v 3r20 3r3q 3r41 3r46 3r6o 3r6t

3r77 3r9r 3ray 3rc1 3rco 3rd7 3rew 3rh4 3rht 3rhz 3rjt 3rl4

3rmq 3ro3 3ro8 3rp8 3rqx 3rtl 3ru6 3ruo 3rxy 3rzi 3s1u 3s1y

3s2z 3s3l 3s80 3s81 3s9d 3s9x 3sd7 3sdu 3sew 3sf6 3sg8 3sgh

3sgw 3sm4 3smp 3smq 3sn1 3sn7 3sns 3so5 3sol 3ssa 3suj 3svi

3sxq 3sz6 3szs 3t2h 3t41 3t6i 3t6l 3t9g 3tbd 3td5 3tee 3tfc

3tfr 3tfs 3tg8 3thc 3ti2 3tiw 3tja 3tjy 3tnl 3tno 3tr9 3trg

3ttg 3tv1 3tx6 3ty1 3tyk 3tyt 3u1d 3u1w 3u3g 3u4m 3u7i 3u9w

3uc7 3ucj 3uf4 3ufe 3ui4 3uia 3umy 3uqa 3us5 3uue 3uuw 3uvc

3uvi 3v0e 3v31 3v4k 3vav 3vbj 3vdh 3vh9 3vig 3vkb 3vmk 3voc

3vot 3vqs 3vrc 3vto 3vty 3vub 3vwd 3vwi 3vzx 3w5f 3w8d 3wjt

3wkg 3wko 3wky 3wmy 3wnf 3zbl 3zbo 3ze9 3zeu 3zfk 3zfp 3zg1

3zj0 3zjb 3zje 3zl1 3zmd 3zn4 3znu 3znv 3zo9 3zoj 3zoq 3zr8

3zvi 3zvl 3zvy 3zw7 3zwt 3zx3 3zxq 4a1u 4a1x 4a1y 4a30 4a57

4a9v 4acf 4adn 4af8 4ah4 4air 4ak5 4amm 4ano 4apx 4aqj 4aru

4at0 4ate 4auv 4aw7 4awe 4ayl 4b0a 4b0h 4b2h 4b41 4b4d 4b4u

4b6z 4b7h 4b8e 4b8x 4b8y 4b96 4b9b 4b9d 4beu 4bg9 4bgu 4bh5

4bjz 4bmc 4bmh 4bmo 4bqn 4brc 4bwc 4bwm 4bwr 4c0z 4c13 4c2o

4c3x 4c81 4c8o 4c9x 4ca1 4cb5 4ch9 4chi 4cxf 4db3 4dbb 4dcc

4ddd 4dev 4df0 4df8 4dfj 4dfr 4dfw 4dgf 4dhd 4dih 4dkk 4doc

4dq6 4dq9 4dr8 4ds3 4dxf 4dxl 4dzi 4e1s 4e38 4e4r 4e69 4e8j

4e8s 4e98 4e9x 4ea9 4eb2 4edp 4eei 4een 4efi 4efz 4egd 4eht

4eib 4eif 4eih 4eiv 4ekv 4eoe 4eps 4eqb 4eqs 4err 4etv 4eu9

4evu 4evy 4ew7 4exl 4ezi 4f0j 4f0s 4f1j 4f2e 4f4d 4f54 4f78

4f8o 4fai 4fb2 4fbl 4fc9 4fcg 4fek 4ffu 4fge 4fj6 4fk7 4fk9

4fkb 4fmy 4fn7 4fp6 4fqg 4fs5 4fs6 4fsd 4fu3 4fxq 4fzl 4g22

4g2k 4g2n 4g3v 4g9b 4g9p 4g9s 4gak 4gbf 4gbm 4gbu 4gci 4gcm

4gd5 4gdx 4ggg 4ghg 4gi5 4gk6 4gkh 4glj 4gm6 4gnr 4gnv 4gof

4gqr 4gqz 4grf 4gsx 4gt9 4gvc 4gvo 4gwb 4gwm 4gx8 4gxw 4gyf

4gyl 4gzc 4h1x 4h2g 4h3u 4h4n 4h59 4h7w 4hat 4hcj 4hdt 4he2

4hen 4heo 4hgf 4hhr 4hhv 4hif 4hjz 4hl1 4hls 4hng 4hps 4hry

4hs1 4hs9 4ht3 4hvk 4hvt 4hw6 4hwc 4hxf 4hzo 4i0w 4i1k 4i3f

4i3m 4i62 4i68 4i90 4i92 4id4 4id9 4ied 4ien 4ifa 4ifs 4igv

4ihu 4ihz 4iik 4iio 4ij5 4ikd 4inc 4inf 4io1 4io2 4iqi 4irt

4iyj 4iyk 4j37 4j3v 4j5r 4j8p 4jbb 4jbe 4jbg 4jcc 4jck 4jd0

4jdp 4jf3 4jg3 4jix 4jja 4jk8 4jll 4jn7 4jo5 4jr2 4jrf 4jti

4jwm 4jwp 4jxk 4jys 4k0d 4k1t 4k22 4k37 4k3f 4k3l 4k4k 4k70

4k7x 4k9z 4ka7 4kem 4kep 4kg7 4kjm 4kkr 4kld 4klf 4klg 4klh

4kli 4klj 4kll 4klm 4klt 4knu 4ktb 4l2i 4l3u 4l4h 4l82 4l9d

4l9o 4l9p 4lc6 4ldv 4ler 4lfs 4lfv 4lgl 4lgy 4lji 4lny 4lpq

4lq6 4lqz 4lrs 4lsb 4lxq 4lzd 4lzg 4m04 4m32 4m3s 4m8k 4m9t

4ma9 4maa 4mai 4mb3 4mby 4mg4 4mge 4mjf 4mmw 4mov 4mpo 4mr0

4mum 4mur 4mx6 4mzg 4mzy 4n5w 4n6d 4n6x 4n7i 4n9s 4nan 4nas

4nck 4ne4 4nmw 4noh 4nsv 4ntd 4ntp 4ntr 4nw4 4nw9 4nx4 4nyh

4nyo 4o0c 4o1r 4o2w 4o5f 4o78 4o9d 4ofk 4og2 4oh0 4oh7 4ol9

4onz 4orf 4ou9 4oyw 4p54 4pq8 4pvk 4pw0 4pxy 4pz0 4pzj
